# Antioxidant Activity as Biomarker of Honey Variety

**DOI:** 10.3390/molecules23082069

**Published:** 2018-08-18

**Authors:** Małgorzata Dżugan, Monika Tomczyk, Patrycja Sowa, Dorota Grabek-Lejko

**Affiliations:** 1 Department of Chemistry and Food Toxicology, Faculty of Biology and Agriculture, University of Rzeszow, Ćwiklińskiej 1a St., 35601 Rzeszów, Poland; mwesolowska@ur.edu.pl (M.T.); patrycjasowa@op.pl (P.S.); 2 Department of Biotechnology and Microbiology, Faculty of Biology and Agriculture, University of Rzeszów, Zelwerowicza 4, 35-601 Rzeszów, Poland; dorobek@o2.pl

**Keywords:** honey, antioxidant activity, phenolics compounds, PCL, cluster analysis, PCA

## Abstract

Honey variety is commonly defined by beekeepers based on nectar flow availability and the only laboratory method to provide verification is the melissopalynological analysis. Therefore, a quick and simple method for accurate assessment of honey variety is still being researched. The aim of the study was to evaluate the antioxidant activity of honey as an indicator of variety through the use of multivariate statistical analysis. Materials for the study consisted of 90 samples of varietal Polish honeys (rape-12, tilia-10, goldenrod-11, dandelion-5, buckwheat-6, multifloral-17, nectar-honeydew-8 and coniferous honeydew-16 and leafy honeydew-5) obtained directly from apiaries. Honeys were investigated in aspect of antioxidant capacity by photochemiluminescence (PCL) methods using standard ACW and ACL kits. As the reference FRAP and DPPH methods were used. The total phenolics content (TPC) was determined through the Folin-Ciocalteu method. The strongest antioxidant activity was found for buckwheat, while the weakest was found for rape honeys regardless of the used method. Results of the used methods were positively correlated (r = 0.42 to 0.94). Analysis conducted by PCL method confirmed that the minor fraction of honey antioxidants exhibits hydrophobic properties. Clear separation of honey varieties using PCA and Clustering method indicate that antioxidant activity can be a useful parameter for determining the botanical origin of honey.

## 1. Introduction

Honey is a natural food product, which next to its nutritional importance, possesses valuable therapeutic properties due to the presence of bioactive ingredients. In general, biologically active compounds in honey can be divided into two groups: Antibacterial and antioxidant [1,2]. However, these two factors affect each other and their combination results in the high health-promoting properties of honey. Honey exhibits a bacteriostatic and bactericidal activity against several human pathogens, especially gram-positive bacteria, such as *Staphylococcus aureus*, *Escherichia coli* and *Pseudomonas* spp [3,4]. The unique antibacterial initiators of honey are: High sugar content, low water activity, hydrogen peroxide, the presence of strong acids, flavonoids and phenolic acids, methylglyoxal and bee defensin-1 [5]. Next to antibacterial activity, honey exhibits strong antioxidant activity. For this property following components are responsible: Polyphenol compounds (phenolic acids and flavonoids), vitamin C, vitamin E, enzymes (e.g., catalase, peroxidase) and trace elements [6]. 

The composition of honey depends primarily on its floral source but seasonal and environmental factors are of great importance [7,8,9]. As a consequence, the chemical composition of honey is extremely variable. Different kinds of honey vary primarily by biological activity, as well as by their chemical composition (volatile compounds, carbohydrates, and phytochemicals), physical properties (color, viscosity, hygroscopic properties and pH) and taste. Therefore, different varieties of honey exhibit different health promoting properties [1,2,10]. For this reason, it is very important to accurately determine honey variety. Currently, beekeepers in most cases determine the variety of honey based on the time of nectar occurrence and the availability of individual nectar flows. The only laboratory method that provides certainty about the honey variety is the melissopalynological method. Such a technique is based on the microscopic quantitative identification of pollen of plants present in the examined honey. Particular difficulties in this method are associated with the need for good experience and knowledge of pollen morphology and the availability of a comprehensive collection of pollen grains. To overcome this problem, and also to save time and money, attempts to predict the botanical origin from some of their physicochemical properties have been tested [11,12,13,14]. Several groups of phytochemical markers of floral origin such as: Volatile compounds, phenolic compounds, carbohydrates, nitrogen containing compounds and microelements have been already investigated [15,16,17].

Among biomarkers phenolic compounds, the main antioxidants reported in honey have been intensively examined. More than 150 phenolic compounds in honey have been investigated, including phenolic acids, and flavonoids [6,18]. These compounds are classified as thermostable honey components and are not sensitive to the effects of elevated temperature [19]. Moreover, it should be noted that instead of antioxidant activity, they exhibit bactericidal, anti-inflammatory, anti-allergenic, anticoagulant and anti-cancer effects [20]. It has been reported that the polyphenols content is significantly correlated with the honey color, indicating that the honey of dark color exhibits a higher content of phenolic compounds, which in turn indicates enhanced antioxidant activity [7,21]. 

For determination of the antioxidant potential of honey, many analytical methods have been developed [22]. The most commonly used assays include DPPH (free radical scavenging activity), FRAP (ferric reducing/antioxidant power), ORAC (oxygen radical absorbance capacity), AEAC (ascorbic acid content), and TEAC (Trolox equivalent antioxidant activity) [23]. Each assay has its advantages and disadvantages. DPPH test is reported to be unaffected by certain side reactions such as metal ion chelation and enzyme inhibition [24]. Furthermore, honey contains abundant free radical scavengers, which are able to reduce the imbalance between free radical production and the antioxidant level [25]. The high amount of reducing sugars in honey (>65%) such as glucose and fructose could contribute to higher reducing antioxidant power in the FRAP assay, which would lead to positive error in the determination of antioxidant activity [26]. In addition, this method is unable to detect slowly-reacting polyphenolic compounds and thiols [27]. The infrequently used photochemiluminescence (PCL) method allows the differentiation of e hydrophobic and hydrophilic antioxidants fractions in regards to their water solubility (water-soluble fraction-ACW test and fat-soluble fraction-ACL test). The main feature of the PCL method is a combination of a simple and reliable process for the production of free radicals and their very sensitive chemiluminometric detection [21,28,29]. 

The aim of the study was to evaluate the antioxidant activity of honey as an indicator of variety with the use of multivariate statistical analysis.

## 2. Results and Discussion

**Figure 1:**
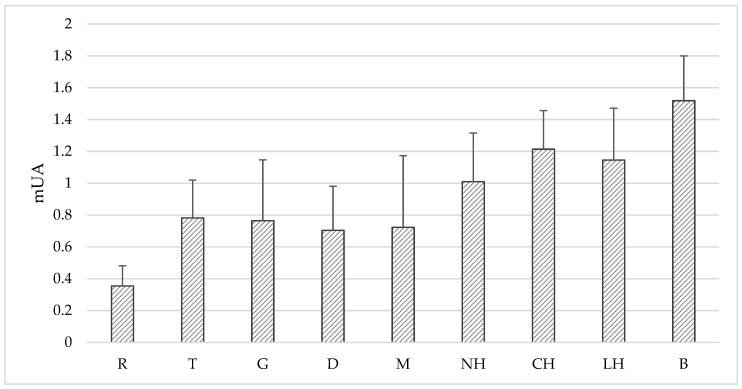
Color intensity of tested honey samples expressed as mAU. Honey variety: R-rape, T-tilia, G-goldenrod, D-dandelion, M-multifloral, NH-nectar-honeydew, CH-coniferous honeydew, LH-leafy honeydew, B-buckwheat. Significant differences (*p* < 0.05): B-R, B-T, B-G, B-D, B-M, R-T, R-NH, R-CH, R-LH.

The quality of studied Polish honeys were examined according to the applicable EU [30] and Polish [31] regulations. All honey samples perfectly met the requirements current standards according to physicochemical parameters (i.e., moisture content, free acidity, pH, electrical conductivity, sugar content, and hydroxymethylfurfural) and sensory parameters (color, odor, taste) (data not shown). Based on the color intensity, tested honeys can be divided into dark-colored honeys exhibiting color intensity over 1 mAU (buckwheat, nectar-honeydew, coniferous honeydew and leafy honeydew) and light honeys exhibiting less than 1 mAU (rape, tilia, goldenrod, dandelion and multifloral) (Figure 1). 

### 2.1. Antioxidant Activity of Tested Honeys

Honey serves as a source of natural antioxidants, which play an important role in food preservation and human health by combating damage caused by oxidizing agents, namely reducing the risk of heart disease, cancer, immune-system decline, cataracts, different inflammatory processes, etc. [32,33,34]. There is no official method for honey antioxidant activity determination and therefore none of the methods used for testing antioxidant activity of honey are ideal, as each of them allows the measurement of a different group of antioxidants. For this reason, in the present study, two standard spectrophotometric methods are used: The DPPH test for radical scavenging activity and the FRAP method for reducing antioxidant power. A novel photochemiluminescence technique (ACW and ACL) was applied (Table 1). Due to the antioxidant and antiradical properties of honey being mainly attributed to the presence of phenolic compounds (Beretta et al., 2005), such components were also discerned in the tested honey samples (Table 1).

Among the honey varieties tested, the greatest variability was observed for goldenrod honey, while rape honey was the most stable. Both melliferous plants have a different flowering period. In Polish conditions, rape blooms about three weeks in May, whereas goldenrod is available for bees in late summer-autumn because such plants are flowering in August–September. This indicates that the more homogeneous nectar the bees collect, the greater the stability of the chemical composition of honey.

**Figure 2:**
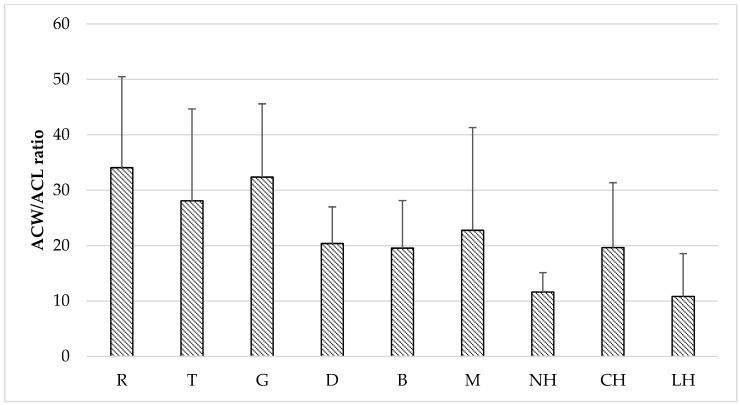
The ratio of antioxidant capacity of hydrophilic to hydrophobic fraction (ACW/ACL) for varietal honey samples determined by PCL assay. Honey variety: R-rape, T-tilia, G-goldenrod, D-dandelion, B-buckwheat, M-multifloral, NH-nectar-honeydew, CH-coniferous honeydew, LH-leafy honeydew

Comparing the results given by different assays, the highest variability can be observed for PCL method results. This method only allows us to differentiate hydrophilic (ACW test) and lipophilic (ACL test) antioxidant fractions in tested honey samples. Studies of the antioxidant capacity of varietal honey samples made by PCL-ACW assay showed different water-soluble antioxidant activity for various types of honey. Significantly, the highest (*p* < 0.05) level of hydrophilic fraction was found for buckwheat and goldenrod honeys (24.03 and 22.77 mM AA kg^−1^, respectively), while the lowest for rape honeys (10.59 mM AA kg^−1^). Among tested samples the lowest (*p* < 0.05) activity of water-insoluble antioxidant fraction (PCL-ACL) was tested for rape honey (0.40 mM TE kg^−1^) as compared to other varieties. Analysis conducted by PCL method confirmed that the minor fraction of honey antioxidants exhibiting hydrophobic properties was the most diverse (VC from 41 to 81%). In the water-soluble fraction, antioxidants such as flavonoids, ascorbic acid and amino acids are detected, while in the lipid soluble fraction tocopherols, tocotrienols and carotenoids are measured [28]. Additionally, calculations of the ACW/ACL ratio showed variation in the composition of the honey antioxidant fraction (Figure 2), but observed differences were not statistically significant (*p* > 0.05). For rape and goldenrod honeys, a strong predominance of water-soluble components was found, while the higher share of this fraction in all kinds of honeydew honeys was detected. Present results are in agreement with our earlier study on 40 varietal Polish honey where we found hydrophilic fractions to be dominating and confirmed statistically significant differences in ACW/ACL ratio dependent on the honey type [21]. 

The antioxidant activity of tested samples was conducted by DPPH assay which is one of the most stable free radical and is frequently used in the evaluation of radical scavengers in natural foods. The average antioxidant activity of tested honey samples measured for 20% *w*/*v* honey solution (expressed as % of inhibition) ranged from 21.81% (rape) to 82.41% (buckwheat) honeys. Obtained results were comparable to the study of Wilczyńska [35], where the DPPH radical reaction system measured for 20% *w*/*v* honey solution varied from 23.8% (Polish nectar-honeydew) to 100% (Polish heather and buckwheat honeys). Jasicka-Misiak et al. [17] described similar values (31–40%) for Polish goldenrod honey measured for 20% *w*/*v* honey solution as compared to the present study. According to Kacaniova et al. [36], the radical scavenging activity of Slovak honeydew honey samples measured for 25% *w*/*v* honey solution varied in the range 45.9–86.6%, similarly to our study. Perna et al. [37] tested Italian honeys and found out the radical scavenging activity measured for 3–60% *w*/*v* honey solution ranges from 55.06% for citrus honey to 75.37% for chestnut honey. DPPH is the easy and simple method; however it is more sensitive to lipophilic antioxidants [38].

Among tested samples, the strongest reducing antioxidant power measured by the FRAP test was found for buckwheat honey (3635.49 μmol TE kg^−1^) which was at least twice as high as in other nectar honeys (*p* < 0.05) and about 40% higher than in honeydew honeys (*p* < 0.05). The lowest reducing antioxidant power was detected in rape honey (656.73 μmol TE kg^−1^). Results are in agreement with other author’s observations, where the levels of antioxidant activity measured by FRAP method ranged from 95 to 2705 μmol TE kg^−1^ [39,40]. On the other hand, results of the present study are significantly higher as compared to Anand et al. [41], who tested Manuka honey and Chua et al. [23] who tested Tulang and Gelam honeys, known in the literature to exhibit strong antioxidant activity [25].

The total phenolics content determined by the modified Folin–Ciocalteu method varied greatly among the honey types, as is apparent from Table 1. However, within single variety this parameter was more stable compared to others and the coefficient of variability was lower than 30%, excluding goldenrod and dandelion honeys. Buckwheat honey was characterized by a significantly higher content of phenolics compounds (on average 1353.66 mg GAE kg^−1^) as compared to other tested varieties (*p* < 0.05). The lowest content of total phenolic compounds was exhibited by rape honey (254.52 mg GAE kg^−1^). For the rest tested varieties, the content of phenolics compounds varied from 409.10 (tilia) to 630.26 (nectar-honeydew) mg GAE kg^−1^ was tested. Obtained results are comparable with other authors’ findings. Wilczyńska [35] found the total phenolics content for Polish honeys in the range from 175.7 (rape) to 1895.2 (heather) mg GAE kg^−1^. In the study of Mellen et al. [42] total phenolics content in multifloral Polish honey ranged from 611 to 990 mg GAE kg^−1^. Bertoncelj et al. [7] tests for Slovenian honeys showed lower values of total phenolics compounds as compared to the present study, varying from 44.8 mg GAE kg^−1^ in acacia honey and rising further in lime, multifloral, forest and honeydew (241.4 mg GAE kg^−1^). 

### 2.2. Statistical Analysis

A strong correlation between the antioxidant activity measured by different methods, other than ACL and ACW, was calculated by Sperman’s rank order (Table 2). Such an observation was also tested in our earlier studies [21,43,44] and has been proved by other authors [7,23,33,45].

#### 2.2.1. Principal Components Analysis (PCA)

PCA was used in several studies to classify different variety of honey as well as to analyze similarities between samples from different floral origins based on chemical composition, physicochemical and antioxidant properties [16,46,47,48,49]. The obtained results confirmed the significant influence of the botanical origin of honey and their chemical composition and other studied parameters. In this work, we focused on evaluation of the differences among honey samples with reference to the antioxidant activity. The classification based on the antioxidants soluble in water and lipids was carried out for the first time. Additionally to antioxidant parameters, color intensity exhibiting strong positive correlation with them (Table 2) was also included during PCA analysis. High correlation between color intensity and antioxidant activity was previously confirmed [7,21,44]. Due to the large number of samples, the average results obtained for each variety of honey were used. Six variables (FRAP, DPPH, TPC, PCL-ACW, PCL-ACL and color) was reduced to the two principal components (PCs). These components accounted for 91.2% of total variance in the analyzed honey samples (PC1 explained 82.81% of variance and PC2 8.42%). According to the loading matrix shown in Table 3, PC1 was strongly negatively correlated with all tested parameters, while PC2 was positively correlated with ACL. This parameter was the most relevant variable for the discrimination of the samples observed on PCA (force 0.98).

**Figure 3:**
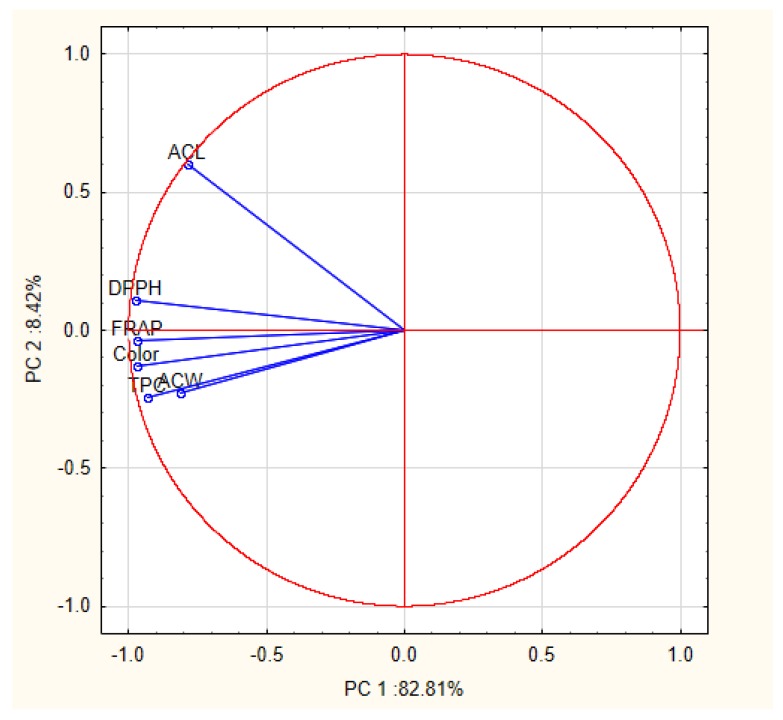
Projection of variables as function of the PC1 vs. PC2.

**Figure 4:**
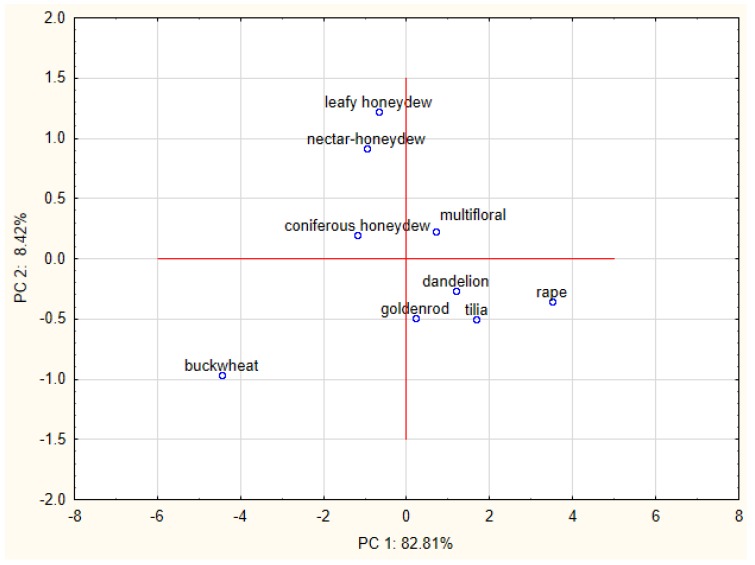
Plot of the PC1 vs. PC2 for classification of studied honey varieties.

The results of PCA analysis were presented on Figure 3 and Figure 4. Variables displayed in Figure 3 show the strongest correlation between ACW and total phenolic content (TPC), but it should be noted that other variables were located in close proximity. It may also suggest that commonly used methods (FPAP and DPPH) are more effective in measuring water-soluble antioxidants, and that phenolics compounds are the main fraction of water-soluble components responsible for antioxidant activity. Figure 4 represents a graphic distribution of studied honey varieties according to their components scores. Varieties of honey which exhibit higher values of antioxidant activity and contain more phenolic compounds such as buckwheat, coniferous honeydew, leafy honeydew and nectar-honeydew (dark honeys) were located on the left side of the plot (negative value of PC1). Light varieties of honeys with lower antioxidant activity were on the right side, with a positive value of PC1. In addition, separation depending on the botanical origin was observed. Honey that was produced mainly from nectar sources were localized at the bottom of the graph (negative value of PC2), and honey obtained from excretions of plant-sucking insects on the living parts of plants (honeydew honeys) in the upper part. The only exception was multifloral honey, which is an extremely diverse honey variety in terms of botanical origin. Such a clear separation of honey varieties using the PCA method indicates that antioxidant activity could be useful parameter for determining the botanical origin of a type of honey.

#### 2.2.2. Cluster Analysis

**Figure 5:**
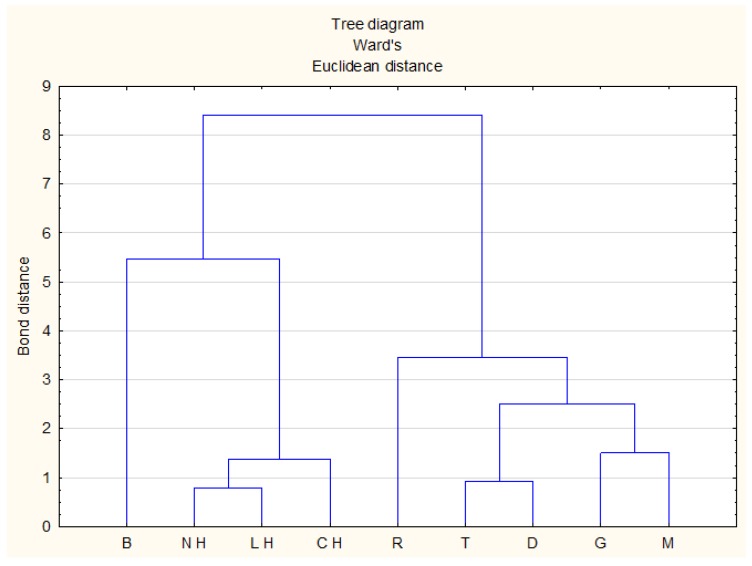
Cluster analysis tree diagram. Honey variety: R-rape, T-tilia, G-goldenrod, D-dandelion, B-buckwheat, M-multifloral, NH-nectar-honeydew, CH-coniferous honeydew, LH-leafy honeydew

Another multivariate method used to classify honey and compare similarities between them based on chemical composition is cluster analysis (CA). In our previous studies, this method was used to find similarities between analyzed groups of honey products (nectar honey, commercial herb honeys, creamed multifloral honey with herb addition and natural herbal honey) based on the average value of antioxidant activity [43,50]. Cluster analysis by the Ward’s method of linkage and Euclidean distance was drawn based on the average value of studied parameters. The results obtained by CA were very similar to the results from PCA analysis. The honey varieties were divided into two main groups (Figure 5). On the left side of the graph there is dark honey with a high value of antioxidant activity, light honey with lower antioxidant activity were placed on the right side. The closed correlation between nectar-honeydew and leafy honeydew as well as coniferous honeydew honey (bond distance 0.8 and 1.3, respectively) was observed. Buckwheat honey was the furthest away, both in CA and PCA analysis. This means that the antioxidant activity of this honey differs significantly from the others. Moreover, rape honey was slightly separated from others (both in CA and PCA analysis) which proves that it has the lowest antioxidant activity among the studied honeys.

## 3. Materials and Methods

### 3.1. Samples

Material for the study consisted of 90 samples of varietal honeys (Table 4) obtained directly from beekeepers operating in southeastern Poland (Podkarpacie, Poland). Honeys were collected in beekeeping season 2016 and were stored in dark at room temperature until the time of analysis, no more than 3 months. The floral origin of samples was specified by beekeepers according to hive location and available floral sources. 

### 3.2. Methods

#### 3.2.1. Antioxidant Capacity PCL Assay

Antioxidant capacity of honey samples was determined by photochemiluminescence (PCL) method using the Photochem^®^ (Analytik Jena AG, Jena, Germany) device according to Wesołowska and Dżugan [21]. The test was performed by two different protocols: Determining the water (ACW) and fat (ACL) soluble antioxidant fractions. Honey solution (10 g L^−1^) in water for ACW and in methanol for ACL was used. Measurements of the total antioxidant capacity were performed using reagent kits provided by the manufacturer (Jena, Germany) strictly according to the manufacturer’s procedure instructions. Results were calculated based on standard curves into mmol of Ascorbic acids equivalents per kg of honey (mM AA kg^−1^) for ACW and μmol of Trolox equivalents per kg of honey (mM TE kg^−1^) for ACL. Results were expressed as mmol Ascorbic acid (AA) kg^−1^ of honey for ACW and μmol Trolox kg^−1^ of honey for ACL. 

#### 3.2.2. DPPH Assay (Radical Scavenging Activity)

Antiradical activity of honey samples was determined using the synthetic free radical 2, 2-diphenyl-1-picrylhydrazyl (DPPH) a method which was assay previously described by Blois [51] with some modification. Honey samples (2 g) were dissolved in 10 mL of distilled water. 0.2 mL of honey solution was mixed with 1.8 mL of 0.1 mM DPPH (Sigma Aldrich Co., St. Louis, MO, USA) solution in methanol (Sigma Aldrich Co., USA) and left in the dark at room temperature for 60 min. Then, the decrease in absorbance was measured spectrophotometrically (UV-VIS Spectrometer Biomate 3, Thermo Sci., Madison, WI, USA) at 517 nm according to methanol as a blank. Trolox (Sigma Aldrich Co., St. Louis, MO, USA) and quercetin (Sigma Aldrich Co., St. Louis, MO, USA) at the concentrations of 0.1–100 µg mL^−1^ in methanol were used as positive control. The radical scavenging activity (A%) was calculated by the following equation:

A% = ((A_0_ − A_a_)/A_0_) × 100

where: A_a_ was the absorbance of the studied sample and A_0_ was the absorbance of the control sample.

#### 3.2.3. FRAP Assay (Total Antioxidant Activity)

The ferric reducing antioxidant power (FRAP) was carried out as previously described by Benzie and Strain [52] modified by Bertoncejl et al. [7]. The FRAP reagent contained 2.5 mL of 10 mM TPTZ (Sigma Aldrich Co, St. Louis, MO, USA) solution in 40 mM HCl, 2.5 mL of 20 mM FeCl_3_ (Sigma Aldrich Co., USA) and 25 mL of 0.3 M acetate buffer (pH 3.6). Aliquots of 0.2 mL of honey solution (1 g 10 mL^−1^) were mixed with 1.8 mL of FRAP reagent and the absorbance of mixture was measured spectrophotometrically (UV-VIS Spectrometer Biomate 3, Thermo Sci., Madison, WI, USA) at 593 nm after 10 min incubation at 37 °C against blank. Calibration curve with linear formula y = 0.026x (R² = 0.998) was prepared for Trolox (Sigma Aldrich Co., St. Louis, MO, USA) ethanol solution at the range 25–300 nmol mL^−1^ and the results were expressed as μmol of Trolox equivalents (TE) per kg of honey (μmol TE kg^−1^ of honey).

#### 3.2.4. Total Phenolic Compounds (TPC)

The determination of the total phenolic compounds (TPC) in honey was performed using Folin-Ciocalteu reagent according method modified by to Pilijac-Zegarac et al. [53]. Aliquots of 0.2 mL of honey solution (1 g 10 mL^−1^) were mixed with 1 mL of 10 % Folin-Ciocalteu reagent (Merck, Darmstadt, Germany) and 0.8 mL of 7.5 % *w*/*v* sodium carbonate (Na_2_CO_3_; POCH S.A., Gliwice, Poland). After incubation at room temperature for 120 min, the absorbance was measured spectrophotometrically (Biomate 3, Thermo, Madison, WI, USA) at 760 nm against blank. TPC was calculated based on calibration curve (y = 0.0555x; R² = 0.998) prepared for gallic acid (Sigma Aldrich Co., St. Louis, MO, USA) at the range 25–250 µg mL^−1^. Results were expressed as mg of gallic acid equivalents (GAE) per kg (mg kg^−1^) of honey.

#### 3.2.5. Color Intensity

Color intensity was determined according to Beretta et al. [33]. 50% (*w*/*v*) aqueous solution of honey were homogenized and centrifuged at 14,000 rpm for five minutes, then the absorbance was measured at 450 and 720 nm using a spectrophotometer Biomate 3 (Thermo, Madison, WI, USA). Color intensity was presented as net absorbance at 450 and 720 nm (mUA).

### 3.3. Statistical Analysis

All assays were done in three repetitions. The results were expressed as mean values with standard deviations (SD). The variable coefficient presented as % (%VC) was also calculated. The significant differences in the level of tested parameters depending on the variety of honey were calculated by one-way analysis of variance followed by Tukey’s (HSD) test (*p* < 0.05). Correlations between tested parameters were established using Spearman’s rank correlation coefficient (r). In order to evaluate the differences among honeys from different botanical origins based on antioxidant properties, total phenolic content and color characteristic multivariate analysis (PCA—Principal component analysis and CA—cluster analysis) was carried out. All calculations were done using software (Cracow, Poland). 

## 4. Conclusions

Polish honeys were characterized by high antioxidant activity compared to products from other countries. Generally, dark honeys showed better antioxidant activity (buckwheat followed by honeydew honeys) as compared to light honeys, except for goldenrod honey, the activity of which was comparable to honeydew honey. The weakest antioxidant activity was exhibited by rape honey, which was 3–6 times lower as compared to buckwheat honey regardless of the applied method. 

The results obtained by various method were positively correlated. The most promising tool to differentiate honey variety PCL method was proposed. Using multivariate statistical analysis (PCA and CA method), the possibility to classify the botanical origin of honey based on antioxidant activity was proved.

## Figures and Tables

**Table 1:** Antioxidant capacity (PCL-ACW, PCL-ACL), reducing/antioxidant power (FRAP), radical scavenging activity (DPPH) for 20% *w*/*v* honey solution and total phenolics content (TPC) of the analyzed honeys depending on their variety. Means ± SD, the range of variability (min-max), and variation coefficient (%VC) were presented.

Honey Variety		PCL-ACW (mM AA kg^−1^)	PCL-ACL (mM TE kg^−1^)	DPPH ** (%inhibition)	FRAP (μmol TE kg^−1)^	TPC (mg GAE kg^−1)^
Rape [R] *n* = 12	mean ± SD	10.59 ± 3.96	0.40 ± 0.19	21.81 ± 3.15	656.73 ± 119.40	254.52 ± 34.71
	min-max	5.37–17.95	0.21–0.73	17.34–27.65	486.54–859.62	205.41–310.81
	%VC	37.43	47.87	14.42	18.18	13.46
Tilia [T] *n* = 10	mean ± SD	12.71 ± 2.06	0.57 ± 0.28	40.53 ± 13.01	1060.19 ± 307.37	409.10 ± 69.76
	min-max	8.29–15.08	0.19–1.22	24.35-65.36	619.23–1626.92	302.70–549.55
	%VC	16.19	49.49	32.11	28.99	17.05
Goldenrod [G] *n* = 11	mean ± SD	22.77 ± 12.46	0.97 ± 0.79	45.34 ± 21.44	1259.97 ± 721.13	456.84 ± 220.20
	min-max	7.01–45.24	0.17–2.32	22.49–82.47	605.77–2350.00	284.68–966.67
	%VC	54.75	80.84	47.27	57.23	48.20
Dandelion [D] *n* = 5	mean ± SD	13.58 ± 4.78	0.76 ± 0.40	42.59 ± 17.65	1593.85 ± 567.98	508.11 ± 180.95
	min-max	7.65–18.98	0.33–1.16	28.36–64.25	1038.46-2257.69	326.13–738.74
	%VC	35.19	52.45	41.44	35.64	35.61
Buckwheat [B] *n* = 6	mean ± SD	24.03 ± 2.68	1.41 ± 0.60	82.41 ± 4.59	3635.49 ± 1328.22	1353.66 ± 314.15
	min-max	19.80–26.88	0.66–2.41	76.42–89.03	1973.08–5051.92	922.52–1876.58
	%VC	11.17	42.51	5.57	36.53	23.21
Multifloral [M] *n* = 17	mean ± SD	16.82 ± 6.07	1.14 ± 0.66	39.89 ± 15.08	1562.67 ± 995.11	490.09 ± 225.30
	min-max	8.74–27.49	0.23–2.40	22.45–65.78	580.77–3340.38	236.94–1021.62
	%VC	36.09	58.07	37.84	63.68	45.97
Nectar-honeydew [NH] *n* = 8	mean ± SD	17.98 ± 7.78	1.62 ± 0.64	59.72 ± 15.19	2013.70 ± 721.08	630.29 ± 170.17
	min-max	10.50–33.44	0.67–2.62	35.26–79.58	911.54–2767.31	409.01–962.16
	%VC	43.28	39.78	25.44	35.81	27.00
Coniferous honeydew [CH] *n* = 16	mean ± SD	19.98 ± 6.08	1.29 ± 0.55	66.82 ± 11.21	2153.37 ± 663.92	600.11 ± 161.52
	min-max	7.83–33.87	0.33-2.17	51.39–85.29	1180.77–3701.92	372.97–1001.02
	%VC	30.57	43.05	16.77	30.83	26.91
Leafy honeydew [LH] *n* = 5	mean ± SD	14.41 ± 4.07	1.62 ± 0.68	61.07 ± 7.87	2019.62 ± 574.85	585.95 ± 166.35
	min-max	8.89–18.60	0.73–2.41	50.47–69.27	1080.77–2448.08	345.95–754.95
	%VC	28.24	41.85	12.89	28.46	28.39
Significant differences *		R-B, R-G, R-CH, T-B, T-G	R-M, R-B, R-NH, R-CH, R-LH, T-LH, T-NH	R-all, B-M, B-D, B-T, B-G, CH-M, CH-D, CH-T, CH-G	B-all, R-M, R-CH, R-LH, R-NH, T-CH	B-all, R-M, R-CH, R-LH, R-NH

* Samples marked with symbols differed significantly (*p* > 0.05) in the columns. ** The positive control for DPPH assay: Trolox at concentration 10 and 50 µg mL^−1^ showed 10.40% and 59.52% of inhibition, respectively; quercetin at concentration 10 and 50 µg mL^−1^ showed 16.51% and 68.69% of inhibition, respectively.

**Table 2:** Correlation of tested methods calculated based on Spearman’s rank order (the level of significance *p* < 0.001).

Variable	PCL-ACW	PCL-ACL	FRAP	TPC	DPPH	Color Intensity
PCL-ACW	1.000					
PCL-ACL	0.422	1.000				
FRAP	0.622	0.673	1.000			
TCP	0.647	0.674	0.943	1.000		
DPPH	0.621	0.648	0.876	0.912	1.000	
Color Intensity	0.597	0.566	0.793	0.831	0.928	1.000

**Table 3:** Component matrix.

Variable	Principal Components (Correlations)	
	PC 1	PC 2
ACW	−0.81	−0.23
ACL	−0.79	0.60
FRAP	−0.97	−0.04
TPC	−0.93	−0.24
DPPH	−0.97	0.11
Color	−0.97	−0.13

**Table 4:** Characteristics of honey samples.

Honey Variety	Number of Samples
Rape (*Brassica napus*)	12
Tilia (*Tilia*)	10
Goldenrod (*Solidago virgaurea*)	11
Dandelion (*Taraxacum officinale*)	5
Buckwheat (*Fagopyrum esculentum*)	6
Multifloral	17
Nectar-honeydew	8
Coniferous honeydew	16
Leafy honeydew	5

## References

[1] Ramanauskiene K., Stelmakiene A., Briedis V., Ivanauskas L., Jakštas V. (2012). The quantitative analysis of biologically active compounds in Lithuanian honey. Food Chem..

[2] Wieczorek J., Pietrzak M., Pomianowski J., Wieczorek Z. (2014). Honey as a source of bioactive compounds. Pol. J. Food Nutr. Sci..

[3] Olaitan P.B., Oe A., Io O. (2007). Honey: A reservoir for microorganisms and an inhibitory agent for microbes. Afr. Health Sci..

[4] Aggad H., Guemour D. (2014). Honey Antibacterial Activity. Med. Arom. Plants.

[5] Kwakman P.H., Zaat S.A. (2012). Antibacterial components of honey. IUBMB Life.

[6] Gheldof N., Wang X., Engeseth N.J. (2002). Identification and quantification of antioxidant components of honeys from various floral sources. J. Agric. Food Chem..

[7] Bertoncelj J., Doberšek U., Jamnik M., Golob T. (2007). Evaluation of the phenolic content, antioxidant activity and colour of Slovenian honey. Food Chem..

[8] Kaškonienė V., Maruška A., Kornyšova O. (2009). Quantitative and qualitative determination of phenolic compounds in honey. Cheminė Technologija.

[9] Manyi-Loh C.E., Ndip R.N., Clarke A.M. (2011). Volatile compounds in honey: A review on their involvement in aroma, botanical origin determination and potential biomedical activities. Int. J. Mol. Sci..

[10] Da Silva P.M., Gauche C., Gonzaga L.V., Costa A.C.O., Fett R. (2016). Honey: Chemical composition, stability and authenticity. Food Chem..

[11] Popek S. (2002). A procedure to identify a honey type. Food Chem..

[12] Terrab A., Diez M.J., Heredia F.J. (2002). Characterization of Moroccan unifloral honeys by their physicochemical characteristics. Food Chem..

[13] Devilliers J., Morlot M., Pham-Dele`Gue M.H., Dore J.C. (2004). Classification of monofloral honeys based on their quality control data. Food Chem..

[14] Khalafi R., Goli S.A.H., Behjatian M. (2016). Characterization and classification of several monofloral iranian honeys based on physicochemical properties and antioxidant activity. Int. J. Food Prop..

[15] Kaškonienė V., Venskutonis P.R. (2010). Floral markers in honey of various botanical and geographic origins: A review. Compr. Rev. Food Sci. Food Saf..

[16] Oroian M., Amariei S., Leahu A., Gutt G. (2015). Multi-element composition of honey as a suitable tool for its authenticity analysis. Pol. J. Food Nutr. Sci..

[17] Jasicka-Misiak I., Makowicz E., Stanek N. (2018). Chromatographic fingerprint, antioxidant activity, and colour characteristic of polish goldenrod (*Solidago virgaurea* L.) honey and flower. Eur. Food Res. Technol..

[18] Tsiapara A., Jaakkola M., Chinou I., Graikou K., Tolonen T., Virtanen V., Moutsatsou P. (2009). Bioactivity of greek honey extracts on breast cancer (MCF-7), prostate cancer (PC-3) and endometrial cancer (Ishikawa) cells: Profile analysis of extracts. Food Chem..

[19] Elbanna K., Attalla K., Elbadry M., Abdeltawab A., Gamal-Eldin H., Ramadan M.F. (2014). Impact of floral sources and processing on the antimicrobial activities of different unifloral honeys. Asian Pacific J. Trop. Dis..

[20] Cornara L., Biagi M., Xiao J., Burlando B. (2017). Therapeutic properties of bioactive compounds from different honeybee products. Front. Pharmacol..

[21] Wesołowska M., Dżugan M. (2017). The use of Photochem device in evaluation of antioxidant activity of polish honey. Food Anal. Method..

[22] Moniruzzaman M., Khalil M.I., Sulaiman S.A., Gan S.H. (2012). Advances in the analytical methods for determining the antioxidant properties of honey: A review. Afr. J. Trad. Complement Altern. Med..

[23] Chua L.S., Lee J.Y., Chan G.F. (2013). Honey protein extraction and determination by mass spectrometry. Anal. Bioanal. Chem..

[24] Amarowicz R., Pegg R.B., Rahimi-Moghaddam P., Barl B., Weil J.A. (2004). Free-radical scavenging capacity and antioxidant activity of selected plant species from the Canadian prairies. Food Chem..

[25] Kishore R.K., Halim A.S., Syazana M.S.N., Sirajudeen K.N.S. (2011). Tualang honey has higher phenolic content and greater radical scavenging activity compared with other honey sources. Nutr. Res..

[26] Ferreira I., Aires E., Barreira J., Estevinho L. (2009). Antioxidant activity of portuguese honey samples: Different contributions of the entire honey and phenolic extract. Food Chem..

[27] Jerkovic I., Marijanovic Z. (2010). Oak (Quercus frainetto Ten.) honeydew honey-approach to screening of volatile organic composition and antioxidant capacity (dpph and frap assay). Molecules.

[28] Besco E., Braccioli E., Vertuani S., Ziosi P., Brazzo F., Bruni R., Sacchetti G., Manfredini S. (2007). The use of photochemiluminescence for the measurement of the integral antioxidant capacity of baobab products. Food Chem..

[29] Zielińska D., Szawara-Nowak D., Michalska A. (2007). Antioxidant capacity of thermally-treated buckwheat. Pol. J. Food Nutr. Sci..

[30] Directive C. (2002). 110/EC of 20 December 2001 relating to honey. Off. J. Eur. Commun..

[31] RMRiRW (2015). Rozporządzenie Ministra Rolnictwa i Rozwoju Wsi z dnia 29 maja 2015 r. Zmieniające Rozporządzenie w Sprawie Szczegółowych Wymagań w Zakresie Jakości Handlowej Miodu (Regulation of the Minister of Agriculture and Rural Development from 29 May 2015 Regarding the Regulation on Specific Requirements in the Field of Commercial Quality of Honey), Dz. U. z 2015, Poz. 850. http://www.dziennikustaw.gov.pl/du/2015/850.

[32] Aljadi A.M., Kamaruddin M.Y. (2004). Evaluation of the phenolic contents and antioxidant capacities of two Malaysian floral honeys. Food Chem..

[33] Beretta G., Granata P., Ferrero M., Faccino F.M. (2005). Standardization of antioxidant properties of honey by a combination of spectrophotometric/fluorimetric assays and chemometrics. Anal. Chim. Acta..

[34] Kücük M., Kolayli S., Karaoglu S., Ulusoy E., Baltaci C., Candan F. (2007). Biological activities and chemical composition of three honeys of different types from Anatolia. Food Chem..

[35] Wilczyńska A. (2010). Phenolic content and antioxidant activity of different types of Polish honey—A short report. Pol. J. Food Nutr. Sci..

[36] Kačániová M., Vukovic N., Bobková A., Fikselová M., Rovná K., Haščík P., Čuboň J., Hleba L., Bobko M. (2011). Antimicrobial and antiradical activity of Slovakian honeydew honey samples. JMBFS.

[37] Perna A., Intaglietta I., Simonetti A., Gambacorta E. (2013). A comparative study on phenolic profile, vitamin C content and antioxidant activity of Italian honeys of different botanical origin. Int. J. Food Sci. Technol..

[38] Prior R.L., Wu X., Saich K. (2005). Standardized methods for the determination of antioxidant capacity and phenolics in foods and dietary supplements. J. Agric. Food Chem..

[39] Gheldof N., Engeseth N.J. (2002). Antioxidant capacity of honeys from various floral sources based on the determination of oxygen radical absorbance capacity and inhibition of in vitro lipoprotein oxidation in human serum samples. J. Agric. Food Chem..

[40] Kesic A., Mazalovic M., Crnkic A., Catovic B., Hadzidedic S., Dragosevic G. (2009). The influence of l-ascorbic acid content on total antioxidant activity of bee-honey. Eur. J. Sci. Res..

[41] Anand S., Pang E., Livanos G., Mantri N. (2018). Characterization of Physico-chemical properties and antioxidant capacities of bioactive honey produced from australian grown agastache rugosa and its correlation with colour and poly-phenol content. Molecules.

[42] Mellen M., Fikselová M., Mendelová A., Haščík P. (2015). antioxidant effect of natural honeys affected by their source and origin. Pol. J. Food Nutr. Sci..

[43] Dżugan M., Sowa P., Kwaśniewska M., Wesołowska M., Czernicka M. (2017). Physicochemical parameters and antioxidant activity of bee honey enriched with herbs. Plant Foods Hum. Nutr..

[44] Sowa P., Grabek-Lejko D., Wesołowska M., Swacha S., Dżugan M. (2017). Hydrogen peroxide-dependent antibacterial action of Melilotus albus honey. Lett. Appl. Microbiol..

[45] Chis A.M., Purcarea C., Dżugan M., Teusdea A. (2016). Comparitive antioxidant content and activity of selected Romanian and polish honey. Rev. Chim..

[46] Sant’Ana L.D., Sousa J.P.L.M., Salgueiro F.B., Lorenzon M.C.A., Castro R.N. (2012). Characterization of monofloral honeys with multivariate analysis of their chemical profile and antioxidant activity. J. Food. Sci..

[47] Nayik G.A., Nanda V. (2015). Physico-chemical, enzymatic, mineral and colour characterization of three different varieties of honeys from kashmir valley of india with multivariate approach. Pol. J. Food. Nutr. Sci..

[48] Kaygusuz H., Tezcan F., Erim F.B., Yildiz O., Sahin H., Can Z., Kolayli S. (2016). Characterization of anatolian honeys based on minerals, bioactive components and principal component analysis. LWT-Food Sci. Technol..

[49] Kek S.P., Chin N.L., Yusof Y.A., Tan S.W., Chua L.S. (2017). Classification of entomological origin of honey based on its physicochemical and antioxidant properties. Int. J. Food Prop..

[50] Purcarea C., Dżugan M., Wesolowska M., Chis A.M., Zagula G., Teusdea A.C., Puchalski C. (2017). A comparative study of metal content in selected polish and romanian honey samples. Rev. Chim..

[51] Blois M.S. (1958). Antioxidant determinations by the use of a stable free radical. Nature.

[52] Benzie I.F.F., Strain J.J. (1996). The ferric reducing ability of plasma (FRAP) as a measure of “Antioxidant Power”: The FRAP assay. Anal. Biochem..

[53] Piljac-Žegarac J., Stipčević T., Belščak A. (2009). Antioxidant properties and phenolic content of different floral origin honeys. J. ApiProd. ApiMed. Sci..

